# First Identification of RNA-Binding Proteins That Regulate Alternative Exons in the Dystrophin Gene

**DOI:** 10.3390/ijms21207803

**Published:** 2020-10-21

**Authors:** Julie Miro, Anne-Laure Bougé, Eva Murauer, Emmanuelle Beyne, Dylan Da Cunha, Mireille Claustres, Michel Koenig, Sylvie Tuffery-Giraud

**Affiliations:** 1 LGMR, Univ Montpellier, 34093 Montpellier, France; julie.miro@inserm.fr (J.M.); anne-laure.bouge@seqone.fr (A.-L.B.); e-murauer@chu-montpellier.fr (E.M.); emmanuelle.beyne@ird.fr (E.B.); dylan.da-cunha@ext.inserm.fr (D.D.C.); Mireille.Claustres@inserm.fr (M.C.); michel.koenig@inserm.fr (M.K.); 2 CHU of Montpellier, 34090 Montpellier, France

**Keywords:** DMD gene, skeletal muscle, RNA-binding proteins (RBPs), alternative splicing, targeted RNA-seq, TDP-43

## Abstract

The Duchenne muscular dystrophy (DMD) gene has a complex expression pattern regulated by multiple tissue-specific promoters and by alternative splicing (AS) of the resulting transcripts. Here, we used an RNAi-based approach coupled with DMD-targeted RNA-seq to identify RNA-binding proteins (RBPs) that regulate splicing of its skeletal muscle isoform (Dp427m) in a human muscular cell line. A total of 16 RBPs comprising the major regulators of muscle-specific splicing events were tested. We show that distinct combinations of RBPs maintain the correct inclusion in the Dp427m of exons that undergo spatio-temporal AS in other dystrophin isoforms. In particular, our findings revealed the complex networks of RBPs contributing to the splicing of the two short DMD exons 71 and 78, the inclusion of exon 78 in the adult Dp427m isoform being crucial for muscle function. Among the RBPs tested, QKI and DDX5/DDX17 proteins are important determinants of DMD exon inclusion. This is the first large-scale study to determine which RBP proteins act on the physiological splicing of the DMD gene. Our data shed light on molecular mechanisms contributing to the expression of the different dystrophin isoforms, which could be influenced by a change in the function or expression level of the identified RBPs.

## 1. Introduction

The Duchenne muscular dystrophy (DMD) gene, the largest human gene spanning 2.2 Mb on the X chromosome, is composed of 79 exons separated by introns whose size varies widely (107 to 248,401 nucleotides). In striated skeletal and cardiac muscle, a full-length transcript of 14 kb encodes dystrophin, a subsarcolemmal 427 kDa rod-shaped protein incorporated into a multimolecular membrane-associated protein complex that connects intracellular cytoskeleton to extracellular matrix. This complex ensures membrane stability during muscle contraction and mediates cellular signaling [1,2]. The dystrophin protein is defective in Duchenne muscular dystrophy (DMD), the most severe and frequent muscular dystrophy in children [3].

The DMD gene exhibits tissue-specific isoform expression regulated by at least seven independent promoters. Three encode the full-length isoforms (Dp427p,m,c), including the Dp427m isoform which drives gene expression in skeletal and cardiac muscles. The products of four alternative promoters in downstream introns encode N-terminally truncated proteins, named according to their molecular weight as Dp260, Dp140, Dp116 and Dp71, that are specifically expressed in various organs [4,5]. The shortest Dp71 isoform is the major DMD gene product in the brain [6,7]. Alternative splicing (AS) further increases the diversity of transcript isoforms expressed from the DMD gene, and some notable differences are seen depending on the tissues. In line with the reported high prevalence of AS in nervous system tissues of vertebrates [8,9,10], splice variants are preponderant in cerebral dystrophin isoforms (Dp427c, Dp140, Dp71), in which they frequently involve the skipping of exons 71, 71–74, and 78 [11,12,13,14]. In contrast, the extent of AS is limited in the muscular Dp427m isoform.

We have recently designed a DMD-targeted RNA-seq procedure to establish more accurately the splicing pattern of the Dp427m isoform in adult skeletal muscle [15]. Our data showed the virtual absence of alternative splicing events (ASEs) in Dp427m, the few events detected (among which were exon 71 and exon 78 skipping) not exceeding 3%. These results suggest that a tight regulation is in place in adult skeletal muscle to maintain the inclusion of all 79 exons in the mature transcript and to produce the full-length dystrophin protein. It is worth mentioning that the penultimate exon 78 undergoes a splicing transition during skeletal muscle development, being spliced out in the embryonic isoform while its inclusion is required for muscle structure maintenance in adult tissue [16,17].

To date, the molecular mechanisms that control the spatiotemporal AS of the DMD gene, as well as the functional role of the generated isoforms, remain largely unexplored. Alternative splicing is an essential process that generates networks of functionally coordinated and biologically important transcript isoforms with tissue specific or developmental stage-specific functions [18,19]. Splicing programs are largely controlled by recruiting RNA-binding proteins (RBPs) that recognize specific regulatory sequences embedded in the pre-mRNAs, thereby promoting or inhibiting spliceosome assembly at adjacent splice sites [20].

This study reports for the first time the identification of RBPs that regulate exon inclusion in the Dp427m isoform. For this purpose, we performed a functional RNAi screen targeting 16 muscle-specific or general RBPs in a normal human muscular cell line. The analysis of splicing changes by the DMD-targeted RNA-seq procedure revealed combinatorial regulation by distinct sets of RBPs of a series of DMD exons. Interestingly, the RBP-responsive exons (PE1a, exons 9, 37, 38, 41, 71, 74, 78) have the particularity of being alternatively spliced in other tissues and/or DMD isoforms. Among them, the two short DMD exons 71 and 78 were the most sensitive to variations in intracellular amounts of RBPs.

## 2. Results

To identify trans-acting factors that play a role in DMD pre-mRNA splicing, we analyzed the splicing changes observed in the Dp427m isoform upon silencing of 16 RBPs by RNA interference (RNAi) in the normal human muscular cell line C25Cl48.

### 2.1. Selection of the Candidate RBPs

The RBPs were mainly selected based on their reported role in the regulation of muscle cell-specific ASEs during myogenic differentiation or developmentally regulated AS transitions [21]. These included the major muscle-specific regulatory factors MBNL1, CELF1, RBFOX1, RBFOX2 proteins [22], RBM4 and PTBP1 known to oppositely modulate muscle-specific exon selection during muscle cell differentiation [23], the STAR Quaking protein (QKI-5) identified as a global splicing factor in muscle cells [24] and the two RNA helicases, DDX5 and DDX17, which contribute to maintain myoblast-specific splicing subprograms [25]. In addition, we selected members of the two main RBP families involved in the regulation of alternative splicing, the serine/arginine-rich proteins (SR proteins) and the heterogeneous nuclear ribonucleoproteins (hnRNPs) [26]. Thus, SRSF1, SRSF2, hTra2ß, hnRNPA1, hnRNPA2/B1, DAZAP1 and the TAR DNA-binding protein 43 (TDP-43), a protein involved in different aspects of RNA processing that was recently shown to play a role in the expression of muscle-specific genes [27,28,29], were included in the functional screen.

### 2.2. Characteristics of the Human Muscular C25Cl48 Cell Line

**Figure 1:**
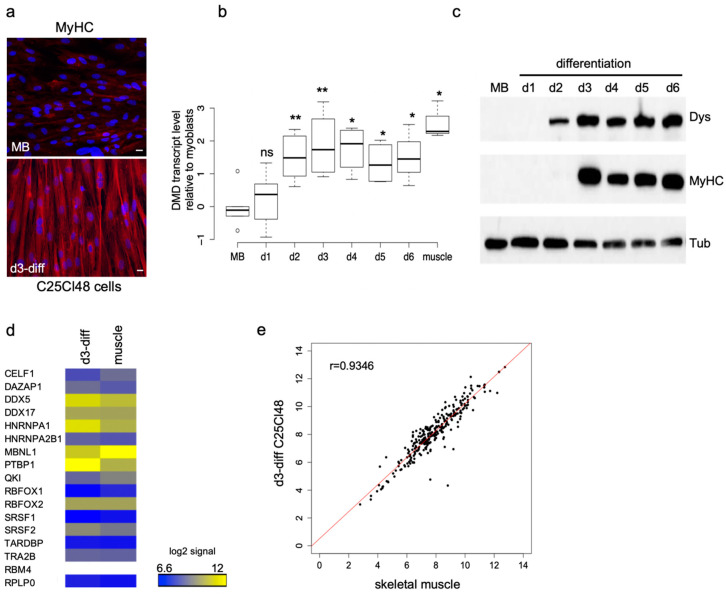
Time-course analysis of gene expression in C25Cl48 cells during myogenic differentiation. (**a**) Visualization of multinucleated myotubes in differentiated C25Cl48 cells. Proliferating myoblasts (MB) and three-days differentiated (d3-diff) C25Cl48 cells were fixed and stained with DAPI (cell nuclei in blue) and with the MY-32 antibody that recognizes all skeletal-muscle myosin (MyHC) isoforms (red). Specific antibody labeling was visualized using a Cy3-conjuguated secondary antibody (laser excitation wavelength of 532–557 nm). Image acquisition was performed using a Leica confocal microscope (20× objective); bar = 15 μm. (**b**,**c**) Duchenne muscular dystrophy (DMD) gene expression in C25Cl48 cells during differentiation (day 1 to day 6) compared with undifferentiated cells (myoblasts, MB) and skeletal muscle tissue. (**b**) Quantitative real-time polymerase chain reaction (RT-PCR) analysis of DMD transcripts levels are normalized on RPLP0 expression and displayed as relative transcript expression to myoblasts. The results are represented as box-and-whisker plots (average of three to six biological replicates for each time point; Wilcoxon rank-sum test ** *p* < 0.01; * *p* < 0.05). (**c**) Western blot analysis of equal amounts of total cellular protein probed with antibodies against dystrophin (Dys) and myosin heavy chain (MyHC) as a differentiation marker. Tubulin (Tub) was used as a loading control. (**d**) Microarray expression profiling of the 16 targeted RNA-binding protein (RBP) genes in C25Cl48 differentiated cells (d3-diff, *n* = 3) and in skeletal muscle samples (muscle, *n* = 3). The heatmap of the replicate-averaged log2 signal intensity of the 16 RBP genes is shown. White boxes are for missing values (RBM4). The expression values for the standard acidic ribosomal protein P0 (RPLP0) gene are displayed. (**e**) Microarray expression profiling of RNA splicing genes (Gene Ontology Term GO:0008380). Scatterplots of replicate-averaged log2 signals of C25Cl48 differentiated cells (d3-diff C25Cl48, *n* = 3) versus skeletal muscle samples (*n* = 3). Red line, best-fit linear regression (*r*, Pearson’s correlation coefficient).

Since the DMD gene is transcribed only at low levels in proliferating myoblasts cultured in vitro [30], we first evaluated the appropriate time point during differentiation to analyze the splicing pattern of Dp427m. After three days of differentiation (d3-diff), the C25Cl48 cells were able to fuse together to form multinucleated myotubes and the expression of the late myogenic marker, myosin heavy chain (MyHC) was detected by immunostaining (Figure 1a). At this time point, Dp427m was efficiently expressed at both the RNA and the protein level in cells (Figure 1b,c). DMD-targeted RNA-seq analysis in d3-diff cells disclosed a basal alternative splicing pattern close to that of skeletal muscle tissue in terms of identity and range of detected ASEs (<5%) [15] except for three events (skipping of exon 71, exon 78 and inclusion of the pseudoexon 1a) that were detected at a higher level in the cells, as previously observed in normal cultured myotubes [31] (Supplementary Table S1). Finally, microarray experiments were performed and showed that the 16 selected RBPs were expressed at a comparable level in skeletal muscle and d3-diff cells except PTBP1 (fold change 3.36) (Figure 1d; Supplementary Table S2). Further analysis of the expression profiles of RNA splicing genes (GO:0008380) revealed a high correlation between d3-diff cells and skeletal muscle tissue (Pearson’s correlation coefficient *r* = 0.93) (Figure 1e, Supplementary Table S2). Considering these preliminary experiments, we chose day three of differentiation as an adequate time point to perform the subsequent splicing analysis of Dp427m in the C25Cl48 cell line.

### 2.3. A Set of DMD Exons Is Sensitive to Changes in Cellular Abundance of RBPs

**Figure 2:**
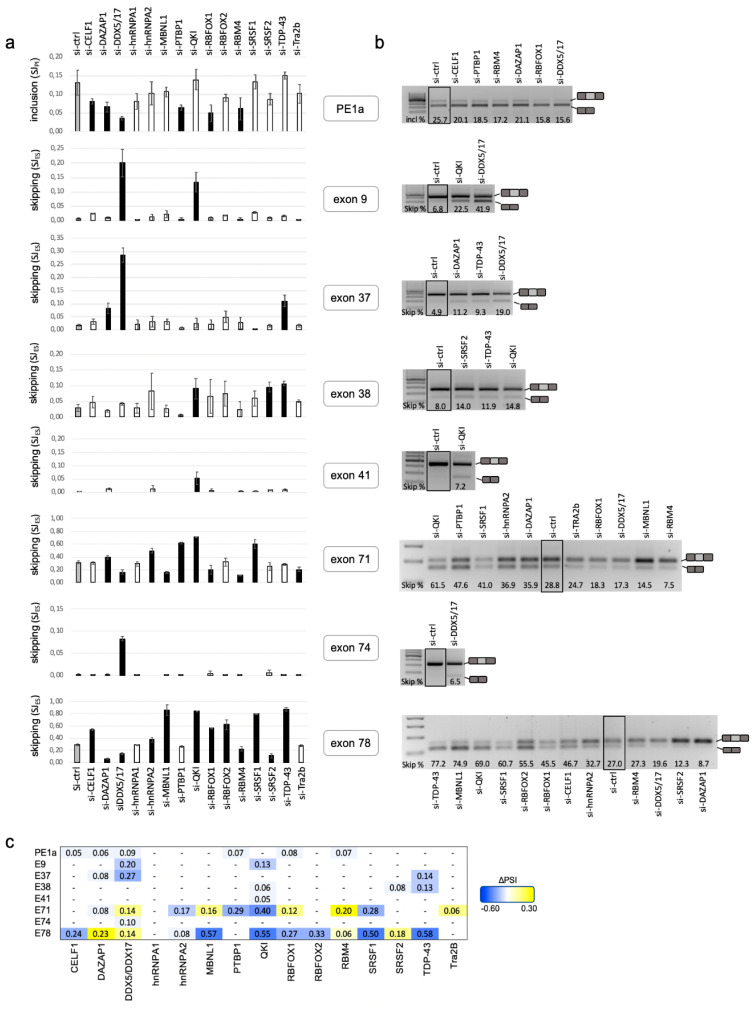
Splicing changes of DMD exons upon depletion of a set of RNA-binding proteins. (**a**) DMD-targeted RNA-seq data. Bar graphs showing the exon skipping level of seven DMD exons (usage of the splice junction (SJ_ES_) resulting from the single exon skipping) or exon inclusion level of the pseudoexon 1a (PE1a) (usage of the splice junctions (SJ_PI_) resulting from the insertion of the pseudoexon) as defined in the Supplementary Figure 2 upon depletion of 16 RBPs in d3-diff C25Cl48 cells. Cells transfected with the control siRNA (si-ctrl, grey bars). Black bars denote the changes in junction usage (ΔSJ = SJ_(si-RBP)_ − SJ_(si-ctrl)_) above the threshold |ΔSJ| ≥ 0.05. Error bars represent the standard deviation from four (si-ctrl) or two (si-RBP) biological replicates. (**b**) RT–PCR validation assay of significative splicing events (|ΔSJ| ≥ 0.05). Agarose gels are shown and the percentages of inclusion (PE1a) or skipping (exons 9, 37, 38, 41, 71, 74, 78) as determined by the Image Lab software are indicated. The control condition (si-ctrl) is in a frame. (**c**) Variations in the inclusion level of the eight deregulated exons calculated from the exon percent-spliced-in (PSI) values provided by the SJPIPE pipeline as follows (|ΔPSI|= |PSI_(si-RBP)_ − PSI_(si-ctrl)_|). Blue boxes (negative ΔPSI values) and yellow boxes (positive ΔPSI values) are indicative of decreased or increased exon inclusion levels upon depletion of RBPs, respectively.

Proliferating C25Cl48 cells were treated with control siRNA or RBP siRNA and then allowed to differentiate for three days. A high level of silencing was generally achieved at both mRNA and protein levels as revealed by the RT-qPCR and Western blot experiments (Supplementary Figure S1). The splicing changes were assessed by DMD-targeted RNA-seq from two (si-RBP) and four (si-ctrl) biological replicates as previously described [15]. Briefly, this approach consisted in amplifying the whole Dp427m 11.3 kb coding sequence by long-range PCR and sequencing on a 454 GS Junior platform. Quality-based trimmed reads (mean length of 374 bp) were mapped to the human X chromosome using the Spliced Transcripts Alignment to a Reference (STAR) aligner. An average read depth of 1638X was obtained, allowing reliable detection of low-level ASEs. The summary of mapping statistics is provided in the Supplementary Table S3. Annotated, as well as novel splice junctions resulting from various splicing events (single or multiple exon skipping, use of alternative 5′ss and 3′ss within exons or introns, inclusion of pseudoexons) were detected. The splice junction (SJ) usage was determined using the intron-centric method [32], and differential analysis was performed to identify splicing alterations between si-RBP- and si-control-treated cells (ΔSJ = SJ_(si-RBP)_ − SJ_(si-ctrl)_). Using a minimum absolute abundance change greater than or equal to 5 percentage points (|ΔSJ| ≥ 0.05), differentially spliced junctions, corresponding to the skipping (SJ_ES_) of 7 DMD exons (9, 37, 38, 41, 71, 74, 78) and the insertion of a pseudoexon (SJ_PI_) originating from intron 1 (PE1a), were identified upon depletion of one or several RBPs. The SJ_ES_ and SJ_PI_ usage reflecting the exon skipping and pseudoexon inclusion levels, respectively, are reported in Figure 2a (see also Supplementary Table S4). All identified ASEs were validated by RT-PCR experiments and the induced exon-inclusion or skipping events detected by the DMD-targeted RNA-seq approach were well correlated with RT-PCR results (Figure 2b). The exon percent spliced in (PSI) index reflecting the global exon inclusion into a population of transcripts was also considered for the 8 RBP-responsive exons. As expected, the changes in inclusion level (ΔPSI = PSI_(si-RBP)_ − PSI_(si-ctrl)_) were very similar to the ΔSJ values consistent with these exons not being spliced out in other major aberrant splicing events (i.e., multiple exons skipping) (Figure 2c, Supplementary Table S4).

Strikingly, the deregulated exons were not random exons but those known to be alternatively spliced in other tissues or dystrophin isoforms [13,33,34] while they are almost fully included (or excluded with respect to PE1a) in the Dp427m isoform of normal skeletal muscle (Supplementary Table S1). Some notable differences were seen among these exons. A subgroup of five exons (9, 37, 38, 41, 74) were sensitive to the depletion of a limited number of RBPs (*n* = 1 to 3) that all induced a decrease in their inclusion levels ranging from 5% to 27% (−0.27 ≤ ΔPSI ≤ −0.05), indicating that these RBPs are required for their inclusion in a physiological context. Regarding PE1a, the depletion of six different RBPs reduced its inclusion level but the changes observed were modest (−0.08 ≤ ΔPSI ≤ −0.05). Exons 71 and 78 differed from the others in that they appeared to be reactive to the depletion of most of the targeted RBPs. The knockdown (KD) of 10 (exon 71) and 12 (exon 78) out of the 16 RBPs influenced positively or negatively the splicing of these two short DMD exons (39 bp and 32 bp, respectively). Except for MBNL1, RBFOX1 and DAZAP1 acting on exons 71 and 78 splicing in opposite directions, the KD of the other RBPs generally induced splicing changes in the same direction on the two exons. We noticed that the depletion of four RBPs induced changes of high magnitude in the inclusion level of exon 78 (|ΔPSI| ≥ 0.5) (MBNL1-, QKI-, SRSF1- and TDP-43-KD) and exon 71 (QKI-KD, ΔPSI = −0.4), thus identifying these RBPs as strong activators of exon 71 or 78 splicing.

The identity and number of regulatory RBPs, as well as their positive or negative activity, varied depending on the deregulated exon. Not considering the peculiar case of PE1a, this study identified a total of seven activators (CELF1, hnRNPA2, PTBP1, QKI, RBFOX2, SRSF1 and TDP-43), two repressors (RBM4 and Tra2ß) of DMD exon inclusion and six proteins regulating splicing in both directions, namely DAZAP1, DDX5/DDX17, MBNL1, RBFOX1 and SRSF2. This functional screen highlighted the more prominent role of QKI and DDX5/DDX17 in DMD pre-mRNA splicing among the tested RBPs as their depletion changed the inclusion level of more than half (four to six) of the eight deregulated exons, acting either as a splicing activator (QKI) or exhibiting opposite effects depending on the deregulated exon (DDX5/DDX17) (Figure 2).

### 2.4. Rare Splicing Events Revealing Differentially Used Deep Intronic Splice Sites

**Figure 3:**
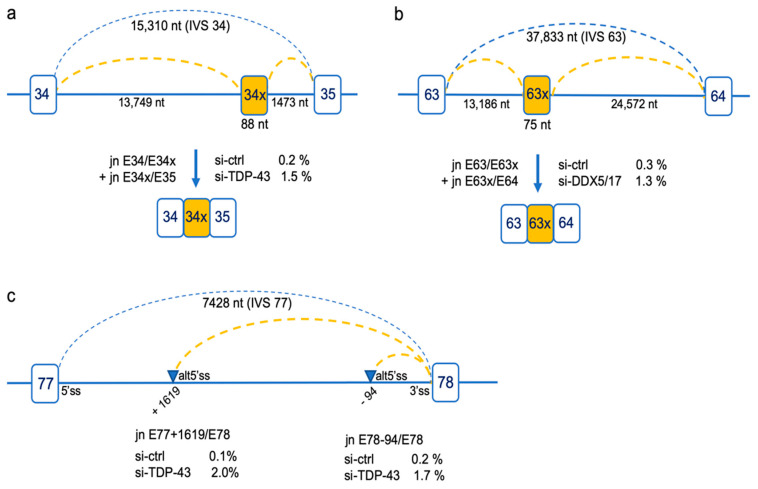
Rare splicing events resulting from the usage of alternative intronic splice sites. When changes in splice junction (SJ) usage in a lower range were considered (0.05 ≥ |ΔSJ| ≥ 0.01), aberrant junctions resulting from the use of deep intronic splice sites were detected in DDX5/17 and TDP-43 KD conditions resulting from the insertion of pseudoexons originating from intron 34 or intron 63 (**a**,**b**) or resulting from the use of alternative 5′splice sites (alt5′ss) in intron 77 (blue triangles) (**c**). The SJ values in RBP (si-TDP-43 and si-DDX5/17) and control (si-ctrl) KD conditions are indicated.

In addition to the differential analyses of SJs based on a cutoff of |ΔSJ| ≥ 0.05, we had a look to less frequent splicing events considering a lower change in junction usage (0.05 ≥ |ΔSJ| ≥ 0.01) (Supplementary Table S5). We observed that other RBPs acted on the inclusion level of exons 9, 37, 38 and 41, albeit to a lesser extent. The skipping of new individual exons (exon 39 and exon 68) and multiple exons (exons 72–74 and exons 71–74) was also detected, the most represented event being the skipping of exon 39 (4.4%) upon DDX5/DDX17 KD. Finally, the analysis disclosed the occurrence of rare splicing events resulting from the use of intronic alternative splice sites. Two were frame-disrupting inclusion of pseudoexons originating from intron 34 (34×) and intron 63 (63×) into the mature transcripts upon depletion of TDP-43 and DDX5/DDX17, respectively (Figure 3a,b). The depletion of TDP-43 was also found to increase the level of splice junctions joining the authentic 3′ss of exon 78 with alternative 5′ss in intron 77 located at positions E77+1619 (2% vs 0.1% in si-ctrl) and E78-94 (1.7% vs 0.2% in si-ctrl) (Figure 3c). These aberrant junctions, present at about 2%, were not modified upon depletion of other RBPs. It is noteworthy that the positions of the 5′ss and 3′ss of pseudoexons 34× and 63×, as well as that of the alternative 5′ss at position E78-1619 in intron 77, are part of the intronic splice sites detected by ultra-deep pre-mRNA-seq of nuclear dystrophin transcripts in a previous study and described as potential recursive splice sites of DMD introns [35]. 

## 3. Discussion

We report the first study aimed at determining which RBPs regulate splicing of the DMD pre-mRNA in skeletal muscle under physiological conditions. We have previously shown that all 79 exons of the Dp427m isoform are constitutively spliced in normal adult skeletal muscle, which is worth mentioning given that this tissue, together with the brain, is known to exhibit extensive AS [18,36,37]. Numerous descriptive studies have depicted tissue-specific ASEs of DMD isoforms, in particular those expressed in the central nervous system [13,14], but the molecular basis of this splicing regulation had remained unexplored to date.

Here, we assessed the role of 16 different trans-acting factors in the splicing of the DMD pre-mRNA, using RNAi in the human C25Cl48 muscular cell line coupled with DMD-targeted RNA-seq of the mature transcript. Targeted RNA-Seq is an accurate method for selecting and sequencing specific transcripts of interest. It is particularly well suited to the analysis of small changes in expression, or in splicing in low-abundance genes such as the DMD gene, owing to the high depth (>1000×) of sequence coverage achieved. The amplicon-based method using specific Dp427m primers efficiently detected splicing variants within the 11.3kb-coding sequence. This method also has inherent drawbacks. It can suffer from PCR amplification biases. Moreover, transcripts with retained intronic sequences whose length exceeds the maximum size limit of amplicons cannot be seen, neither those resulting from the use of alternative poly-A sites [16,38]. Nevertheless, this experimental procedure proved successful in identifying ASEs induced by the KD of the 16 RBPs and all DMD-targeted RNA-seq data were thoroughly validated by RT-PCR and Sanger sequencing.

Interestingly, it was noted that the depletion of the targeted RBPs did not change the inclusion level of constitutive exons but selectively affected exons that undergo spatio-temporal alternative splicing in the various DMD isoforms. These are all in-frame skipping events except the inclusion of PE1a harboring an in-frame stop codon, which may target the resulting transcripts for nonsense-mediated decay (NMD) when included. The inclusion of this cryptic NMD-exon, originating from intron 1 close to the transcription start site, may function as a negative feedback control of DMD gene expression in these tissues or cells as recently described for noncanonical splicing events [39]. The inclusion of PE1a is a very constant phenomenon in all nonmuscle tissues, notably in Dp427m transcripts ectopically expressed in peripheral blood lymphocytes or in the brain Dp427c isoform in neuronal cells [13,33]. Like PE1a, the DMD exon 9 is skipped in many nonmuscle tissues [34]. In-frame splicing changes are more likely to produce functionally relevant isoforms in tissue-specific contexts. Exon 9 skipping removes amino acid residues 278–320 in the first of the four flexible hinge regions that are interspersed in the midrod domain of dystrophin [40]. Alternative splicing of central DMD exons has been described in neuronal SH-SY5Y cells, in which the mutually exclusive skipping of exons 37 and 38, and the activation of an upstream cryptic 3′ss for exon 41 creating a novel enlarged exon 41, have been identified [13]. We did not detect the usage of this cryptic 3′ss in intron 40 upon RBPs KD in the muscular cell line, but a low-level of exon 41 skipping was observed upon depletion of QKI. Splicing variants at the 3′ end of the gene characterize DMD transcripts in the central nervous system. They consist in combinations of single or multiple exon skipping involving exons 71 to 74 and exon 78, with the isoforms lacking exon 71 and/or exon 78 being by far the most represented [12,13,16,41,42,43]. In Dp71, the splice isoforms determine the differential subcellular localization and various cellular functions of the short dystrophin isoform in the brain [44,45]. Transcripts spliced out for exon 78 produce a protein with an elongated C-term region by replacing the last 13 amino acids in the protein with an evolutionary conserved sequence of 31 new residues encoding the ancestral alternative amphipathic C-terminal α-helix.

The inclusion of exon 78 is also regulated during development in the brain and in skeletal muscle [41,46]. The proportion of transcripts lacking exon 78 falls dramatically as development progresses, and MBNL1 has been shown to regulate this splicing transition in skeletal muscle [17]. Experiments in zebrafish and mdx mouse models have confirmed that the reinclusion of exon 78 is essential for skeletal muscle development and muscle fiber organization in adult tissues. The reversion to neonatal splicing pattern of exon 78 (transcripts E78-), due to the sequestration of nuclear MBNL1 and loss of function of the protein, contributes to the progressive dystrophic process in patients with myotonic dystrophy type 1 [17]. In the DMD gene, only one case of single genomic deletion of exon 78 has been reported in a mildly affected 13-year-old BMD patient in whom only minimal impact on morphology and architecture of muscle fibers was observed [47]. Our data add significant new knowledge about splicing regulation of exon 78 by identifying new major regulators of its inclusion in the Dp427m isoform in addition to the previously known enhancer role of MBNL1 that was confirmed here. These are mainly QKI, SRSF1 and TDP-43 and to a lesser extent CELF1, RBFOX1 and RBFOX2. We observed some low-level cryptic splicing events associated with TDP-43 depletion (inclusion of cryptic exon or switching from a canonical splice site to an alternative one), particularly in intron 77. This observation is worth mentioning in light of the recently described role of TDP-43 in repressing cryptic exons and maintaining intron integrity [48,49] although the rarity of the events observed does not allow us to firmly establish this role for the DMD gene.

A remarkable finding is the large number of RBPs found to be involved in splicing regulation of exon 71 and exon 78, the two shortest exons of the gene. To some extent, these two exons share some characteristics with microexons [50]. They meet the least restrictive definition of size (6–51 nt) [51], their splicing is developmentally and/or tissue-specifically regulated, they are frame-preserving or compatible with the synthesis of a stable protein isoform and assumed to be functional as part of protein complexes. A high density of intronic enhancing signals would compensate for the difficulty of the splicing machinery to be efficiently recruited on such short exons [52]. The high number of RBPs identified as splicing modulators of the DMD exons 71 and 78 may reflect this complex interaction between numerous regulatory sequences.

In conclusion, this study uncovered RBPs that are important for the inclusion in the muscular dystrophin isoform of a subset of DMD exons, highlighting more specifically the role of QKI and the RNA helicases DDX5/DDX17, known global regulators of alternative splicing during muscle development and cell differentiation [21,22,24,25]. The next challenge will be to dissect the molecular mechanisms underlying this regulation. Our findings form a valuable basis for further investigations to evaluate whether changes in the amount or activity of the identified RBPs contribute to splicing of the DMD gene in different cells, tissues or developmental stages. This is of particular interest regarding the cerebral dystrophin isoforms, which play a crucial role in the development and function of the human brain and are coexpressed with genes implicated in neurodevelopmental disorders [53]. DMD affects the central nervous system in about one-third of cases, mostly due to distal mutations affecting the expression of the shorter cerebral isoforms (Dp140, Dp71) [54,55]. However, the pathological mechanisms of CNS involvement have not been fully elucidated, notably in patients in whom the expression of cerebral dystrophin isoforms is preserved. This study paves the way for future work aimed at better understanding the regulation of dystrophin isoforms expression in relation to the coordinated splicing networks in tissues in physiological or pathological conditions.

## 4. Materials and Methods

### 4.1. Cell Culture

The human immortalized myoblast cell line C25Cl48 established from a muscle biopsy of a 25-years-old healthy individual [56] was cultivated as previously described [57]. The differentiation was induced by serum deprivation and elevation of insulin concentration to 10 µg/mL.

### 4.2. siRNA-Mediated Depletion of RBPs

Two consecutive siRNA transfections at a 48-h interval were performed. The first day C25Cl48 cells were seeded at 1.4.10^5^ cells per well in six well-plates and reverse transfected with 37.5 nM of individual siRNA or 20 nM siRNA for CELF1, MBNL1 and QKI, using 7.5 µL lipofectamine RNAiMax (Thermo Fischer Scientific, Illkirch, France) following the manufacturer’s instructions. Si-RBP transfections were performed in duplicate. The siGENOME nontargeting siRNA#5 (Dharmacon, Lafayette, CO, USA) was used as a negative control (*n* = 4). The differentiation was induced for three days from the day of the second siRNA treatment, then cells were harvested for protein and RNA isolation. The target sequences and origin of the siRNAs used are described in Supplementary Table S6. Knockdown was validated by RT-qPCR and Western blot analysis.

### 4.3. DMD-Targeted RNA-Seq

#### 4.3.1. Sample Preparation

Total RNA was extracted using the RNeasy plus mini kit (Qiagen, Courtaboeuf, France). cDNA was prepared from 300 ng total RNA using 2.5 µM oligo dT and Superscript II (Thermo Fisher Scientific, Illkirch, France) as described by the manufacturer.

#### 4.3.2. Long Range-Polymerase Chain Reaction (LR-PCR) and 454-Roche Library Construction and Sequencing

DMD-targeted RNA-seq was performed as previously described [15]. Briefly, the full-length DMD cDNA was amplified as a single long fragment (11.3 kb) by using the GoTaq^®^ Long PCR Master Mix (Promega, Charbonnières-les-Bains, France) and primers located in exon 1 and in the 3′ UTR of the muscle isoform (Dp427m). The library was prepared from 700 ng of purified LR-PCR products using the Rapid Library Preparation Kit (Roche, Basel, Switzerland). Three Multiplex Identifiers (MID)-tagged samples were pooled for simultaneous amplification and sequencing on the Roche GS Junior 454 sequencer (Roche, Basel, Switzerland).

#### 4.3.3. Bioinformatics Analysis

Cleaned reads were aligned against the GRCh37/hg19 human X chromosome reference sequence using the STAR aligner (v. 2.3) [58]. Quantification of ASEs was performed based on intron-centric metrics using the integrative pipeline for splicing analyses (IPSA) package [32]. Only the reads that aligned to splice junctions, and not to exon bodies, were considered. From the output file with junction counts (SJcount) noncanonical, novel splice junctions (SJs) covered by a minimum of five reads in at least one out of the two biological replicates of siRBP-treated cells were selected. The identified SJ resulted from the occurrence of exon skipping (ES), pseudoexons insertion (PI) or use of alternative splice site (Altss) events in RBP knockdown conditions. Two separate indices, psi5 and psi3, provided by the SJPIPE pipeline for each SJ were used to calculate the SJ usage resulting from exon skipping (SJ_ES_), pseudoexon insertion (SJPI) or alternative splice site (SJAltss) (details in the calculation method are given in the Supplementary Figure S2). The change in junction usage (ΔSJ) was determined as follows ΔSJ = SJ_(si-RBP)_ − SJ_(si-ctrl)_ using the mean SJ values of the replicates (si-RBPs, *n* = 2 and si-ctrl, *n* = 4). Values greater or equal to five percentage points (|ΔSJ|=|SJ_(si-RBP)_ − SJ_(si-ctrl)_| ≥ 0.05), and not overlapping standard deviation with the control condition, were considered differentially spliced. The exon inclusion metrics or percent-spliced-in (PSI) ratio were also recovered from the SJPIPE pipeline for each individual deregulated exon and the mean values of biological replicates were then used to calculate the change in exon inclusion as follows: ΔPSI = PSI_(si-RBP)_ − PSI_(si-ctrl)._

#### 4.3.4. Availability of Data

*DMD*-targeted RNA-seq data for the RBPs silencing experiments were deposited in the gene expression omnibus repository (accession number GSE153803). 

### 4.4. RT-PCR Validation and RT-qPCR

To validate RNA-seq data, independent RT reactions were performed from RBP-depleted and si-ctrl RNA samples using random primers and 300 ng of total RNA. For RT-PCR experiments, 1.5 µL of cDNA was used as a template for PCR amplification in a 25 µL total volume with the Taq DNA polymerase (New England Biolabs, Evry, France) and primers hybridizing to upstream and downstream exons (sequences available in Supplementary Table S6). The 30 cycles-amplified products were separated on 1.8% agarose gels and spliced products were quantified with ImageLab software (Bio-Rad, Marnes-la-coquette, France). The identity of each amplified PCR product was verified by Sanger sequencing. For quantitative PCR analysis, 1 µL of 1/3-diluted cDNA was amplified using SYBR Green I Master kit in a final volume of 10 µL containing 0.5 µM of gene-specific primers (Supplementary Table S6) on a Light Cycler 480 II (Roche, Basel, Switzerland). For each biological replicate of siRNA-treated cells, two independent reverse transcription reactions were carried out and qPCR assays were run in triplicate and included no-template controls for each siRNAs. Expression levels were calculated according to the 2−∆∆Ct method normalized to RPLP0 expression (Hs_RPLP0_2_SG QuantiTect Primer Assay, Qiagen, Courtaboeuf, France). For assessment of the Dp427m transcript level, a forward primer spanning the exon1-exon2 junction (5′-TAGAGGACTGTTATGAAAGAGAAG-3′) and a reverse primer in exon 4 (5′-CAGGGCATGAACTCTTGTG-3′) were used.

### 4.5. Western Blotting

Cells were washed twice with PBS and lysed in Laemmli sample buffer supplemented with 5% β-mercaptoethanol except for SRSF2 analysis. SRSF2 solubilization required incubation in RIPA buffer supplemented with protease inhibitor cocktail (Sigma-Aldrich, St Quentin, France) during 30 min on ice and under agitation, then samples were centrifuged (14,000 rpm) at 4 °C for 15 min and the supernatant was diluted in Laemmli sample buffer supplemented with 5% β-mercaptoethanol. The samples were subjected to sonication prior to loading into 10% sodium dodecyl sulphate (SDS)-polyacrylamide gels. The proteins were transferred to polyvinylidene fluoride (PVDF) membranes and probed using specific antibodies against CUG-BP1 (3B1, sc-20003, Santa-Cruz, Heidelberg, Germany), DAZAP1 (gift from F. Pagani, Trieste, Italy), DDX5 (ab10261, Abcam, Paris, France), DDX17 (ab24601, Abcam), hnRNPA1 (4B10, sc-32301, Santa-Cruz, Heidelberg, Germany), hnRNPA2/B1 (DP3B3, sc-32316, Santa-Cruz), MBNL1 (MB1a, gift from G. Morris, CIND, Oswestry, UK) [59], PTBP1 (PTB-NT 4856) and RBFOX1 (1D10) (gifts from Dr D. Black, UCLA, LA, USA), QKI (A300-183A, Bethyl, Hamburg, Germany), RBFOX2 (A300-864A, Bethyl, Hamburg, Germany), RBM4 (E-3, sc-373852, Santa-Cruz, Heidelberg, Germany), SRSF1 (32-4500, Thermo Fischer Scientific, Illkirch, France), SRSF2 (PA5-12402, Thermo Fischer Scientific, Illkirch, France), TDP-43 (G400, Ozyme, Saint-Cyr-l’École, France). Anti-ß-Tubulin (AA2, Millipore, Molsheim, France) was used as loading control. The blots were developed using an enhanced chemiluminescence (ECL) kit (Pierce, Rockford, IL, USA). For gene expression analysis during myogenic differentiation, antibodies against dystrophin (MAB1694, Millipore, Molsheim, France) and fast skeletal myosin (My-32, Sigma-Aldrich, St Quentin, France) were used.

### 4.6. Microarray Analyses

For microarray analysis, total RNA samples from three human skeletal muscles (Myobank 20316, Clontech ref#636534, lots #1404229A and #1406360A) and three biological replicates of C25Cl48 cells differentiated at day three were processed, hybridized on a GeneChip^®^ Human Gene 2.0 ST Array, scanned, and quantified at the Affymetrix Service Provider and Core Facility, KFB—Center of Excellence for Fluorescent Bioanalytics (University of Regensburg, Regensburg, Germany; www.kfb-regensburg.de).

## Supplementary Materials

Supplementary materials can be found at https://www.mdpi.com/1422-0067/21/20/7803/s1. Table S1: The alternative Splicing Events (ASEs) detected by DMD-targeted RNA-Seq in C25Cl48 cells differentiated for 3 days; Table S2: The gene expression microarray data in d3-diff C25Cl48 cells and in skeletal muscle; Table S3: The Quality assessment of targeted RNA-seq data; Table S4: The DMD-targeted RNA-seq data: differential usage of splice junctions |ΔSJ| ≥ 0.05; Table S5: The DMD-targeted RNA-seq data: differential usage of splice junctions 0.05 ≥ |ΔSJ| ≥ 0.01; Table S6: The lists and sequences of siRNAs and primers used in this study; Figure S1: The assessment of siRNA-induced silencing of gene expression of the selected RNA-Binding Proteins (RBPs); Figure S2: The calculation of novel Splice Junction (SJ) usage according to the intron-centric metrics method.
