# Supplementary material for: First Identification of RNA-Binding Proteins That Regulate Alternative Exons in the Dystrophin Gene
Source: Int J Mol Sci. 2020 Oct 21;21(20):7803. doi: 10.3390/ijms21207803 (PMC7589424; doi:10.3390/ijms21207803)
Supplement: Supplementary file 1 [file ijms-21-07803-s001.zip › supplemental data/Suppl Table S1.pdf]

**Supplementary Table 1**

| Alternative Splicing Event (ASE) | C25Cl48 d3-diff (%) | skeletal muscle[a] (%) | predicted impact on the reading frame |
|---|---|---|---|
| del E71 | 37.32 | 1.80 | in frame |
| del E78 | 27.30 | 2.48 | elongated C-term |
| PE1a | 12.69 | 3.06 | out of frame |
| del E38 | 4.93 | ND | In frame |
| del E37 | 2.97 | 0 | In frame |
| del E6-41 | 2.18 | 0 | in frame |
| 3’ss exon 54 (-3 nt) | 1.75 | 1.12 | in frame |
| del E9 | 1.55 | 0.63 | in frame |
| del E2 | 1.01 | ND | out of frame |
| PE51X | 0.56 | 0.21 | in frame |
| del E71-74 | 0.42 | ND | in frame |
| del E39 | 0.43 | ND | in frame |
| 3’ss exon 78 (-4 nt) | 0.26 | 0.43 | out of frame |
| del E17-44 | 0.33 | 0 | in frame |
| del E41 | 0.38 | ND | in frame |
| del E73 | 0.28 | 0.32 | in frame |
| del E19-44 | 0.27 | 0 | in frame |
| del E56-78 | 0.26 | 0 | in frame |
| 3’ss exon 76 (-60 nt) | 0.32 | 0.33 | in frame |
| del E68 | 0.25 | ND | out of frame |
| PE12X | 0.25 | 0 | in frame |
| PE2a | 0.12 | ND | out of frame |
