# Supplementary material for: First Identification of RNA-Binding Proteins That Regulate Alternative Exons in the Dystrophin Gene
Source: Int J Mol Sci. 2020 Oct 21;21(20):7803. doi: 10.3390/ijms21207803 (PMC7589424; doi:10.3390/ijms21207803)
Supplement: Supplementary file 1 [file ijms-21-07803-s001.zip › supplemental data/Legend_to_suppl_Tables.pdf]

Legends to Supplementary Tables

Table S1 Alternative Splicing Events (ASEs) detected by *DMD*-targeted RNA-Seq in C25Cl48 cells differentiated for 3 days. List of ASEs in descending order of the percentage detected by DMD-targeted RNA-seq in C25Cl48 cells at day 3 of differentiation (d3-diff) and their respective values in skeletal muscle as reported in (a) (Bougé et al. 2017). In these experiments (four biological replicates each), only new junctions covered by a minimum of 5 reads in at least 2 out of the 4 replicates were considered. ND denotes "not determined" when the minimum filtering criteria (new junction with ≥5 reads in at least 2/4 of the replicates) were not reached but the number of reads is different from zero. The values of Alternative Splicing Events (ASEs) are derived from the Splice Junction (SJ) usage provided by the SJPIPE pipeline for pseudoexon (PE) inclusion (ASE (%)= SJ_{PI}x100), for exon skipping events (del) (ASE (%)=SJ_{ES}x100) and for use of alternative 3' splice sites (3'ss) (in brackets, number of exonic nucleotides deleted when the alternative 3'ss is used) (see Supplementary Figure S2 and Materials and Methods for more details of the calculation).

Table S2 Gene expression microarray data in d3-diff C25Cl48 cells and in skeletal muscle. For microarray analysis, total RNA samples from three human skeletal muscles (Myobank 20316, Clontech ref#636534, lots #1404229A and #1406360A) and three biological replicates of C25Cl48 cells differentiated at day 3 were processed, hybridized on a GeneChip® Human Gene 2.0 ST Array, scanned, and quantified at the Affymetrix Service Provider and Core Facility, ''KFB – Center of Excellence for Fluorescent Bioanalytics'' (University of Regensburg, Regensburg, Germany; www.kfb-regensburg.de). Briefly, RMA normalization and Student's *t*-test were used for statistical analysis. Benjamini–Hochberg correction for

multiple testing was applied in all tests and corrected P-values (FDR) <0.05 were considered significant.

GO 0008380 sheet: gene expression microarray data for the 305 genes analysed related to RNA splicing (GO:0008380). The 18 genes with a up- (log2 fold-change of expression level (FC >2)) or down- (FC<-2) regulation in d3-diff C25Cl48 cells *versus* skeletal muscle tissue (False Discovery Rate (FDR)<0.05, two-Sample Student's t-tests) are highlighted in a different color.

FDR<0.05 and FC<-2 or >2 sheet: list of the genes that display statistically significant regulation in d3-diff C25Cl48 cells *versus* skeletal muscle tissue (FC >2 or FC<-2, FDR<0.05, two-Sample Student's t-tests). Over the 31,650 coding transcripts that were explored, a total of 2,605 (8.2 %) corresponding to 2,515 genes showed differential regulation, of which, 1,557 were up- (FC >2) and 958 were down- (FC <-2) regulated.

Table S3 Quality assessment of targeted RNA-seq data.

RNA-seq read mapping sheet: the total number of reads mapping to the X-chromosome is provided for each sample as well as uniquely mapped, multi-mapped and unmapped reads.

RNA-seq read counts sheet: the average read depth calculated from the total number of uniquely mapped reads and the average mapped length obtained for each sample is given.

Table S4 DMD-targeted RNA-seq data: differential usage of splice junctions $|\Delta SJ| \geq 0.05$.

$|\Delta SJ| \geq 0.05$ sheet: data from STAR mapping (reads) and Integrative Pipeline for Splicing Analyses (IPSA) package (psi5, psi3) are given for each Splice Junction (SJ) found differentially used at more than 5 % ($|\Delta SJ| \geq 0.05$) between the si-RBP and si-control conditions. Data are given for each replicate of the si-ctrl (#1 to #4, line highlighted in grey) or the si-RBP (#1 and #2) treated cells. The usage of the SJ is determined by the mean of psi5 and

psi3 values (see Supplementary Figure S2 for the calculation details depending on the type of splicing events). Change in SJ usage is calculated as follows: $\Delta SJ = SJ_{(si\text{-}RBP)} - SJ_{(si\text{-}ctrl)}$.

PSI exon sheet: Percent spliced in index (PSI) of RBP-responsive exons. The PSI values provided by the IPSA pipeline indicates the efficiency of splicing a specific exon into the transcript population of a gene (exon relative usage). Change in exon usage between conditions are calculated as follows: $\Delta PSI = PSI_{(si\text{-}RBP)} - PSI_{(si\text{-}ctrl)}$.

Table S5 DMD-targeted RNA-seq data: differential usage of splice junctions $0.05 \geq |\Delta SJ| \geq 0.01$. Data from STAR mapping (reads) and Integrative Pipeline for Splicing Analyses (IPSA) package (psi5, psi3) are given for rare splicing events corresponding to differentially used Splice Junction (SJ) in the interval of $0.05 \geq |\Delta SJ| \geq 0.01$ between the si-RBP and si-control conditions. Data are given for each replicate of the si-ctrl (#1 to #4, line highlighted in grey) or the si-RBP (#1 and #2) treated cells. The usage of the SJ is determined by the mean of psi5 and psi3 values (see Supplementary Figure S2 for the calculation details depending on the type of splicing events). Change in SJ usage is calculated as follows: $\Delta SJ = SJ_{(si\text{-}RBP)} - SJ_{(si\text{-}ctrl)}$.

Table S6 Lists and sequences of siRNAs and primers used in this study.
