# Supplementary material for: First Identification of RNA-Binding Proteins That Regulate Alternative Exons in the Dystrophin Gene
Source: Int J Mol Sci. 2020 Oct 21;21(20):7803. doi: 10.3390/ijms21207803 (PMC7589424; doi:10.3390/ijms21207803)
Supplement: Supplementary file 1 [file ijms-21-07803-s001.zip › supplemental data/Supplementary Figures.pdf]

Supplementary Fig. S1

a

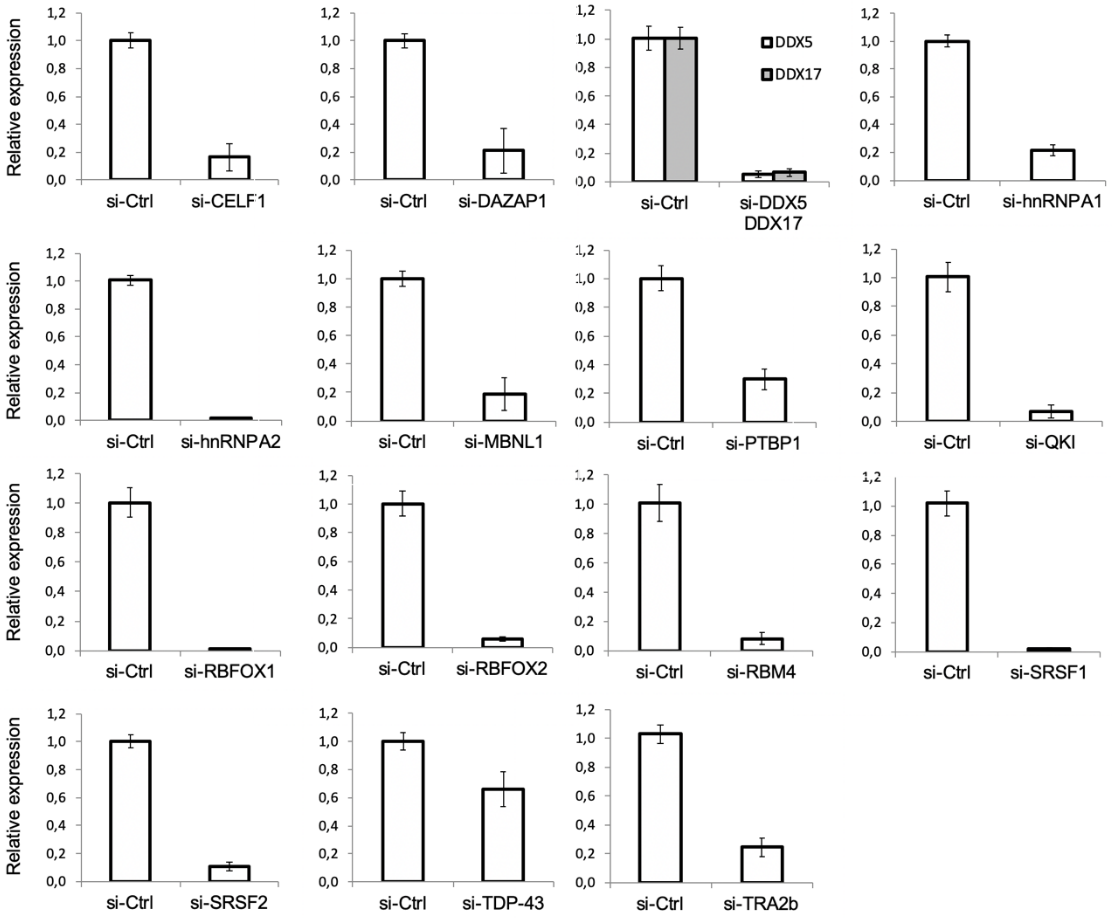

b

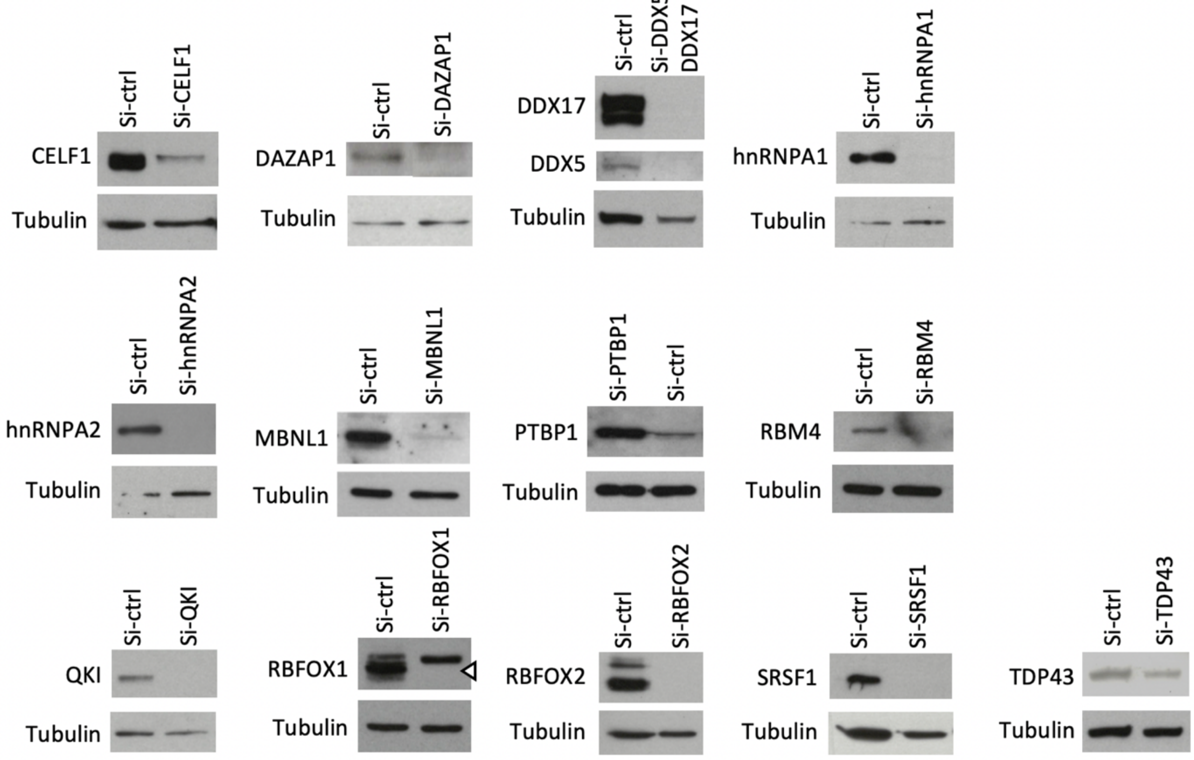

Supplementary Fig. S1 Assessment of siRNA-induced silencing of gene expression of the selected RNA-Binding Proteins (RBPs). RT–qPCR analysis of the relative mRNA level of RBPs in d3-diff C25Cl48 cells transfected with either the control negative siRNA (si-Ctrl) or the specific siRNA showing a generally marked decrease of the target RBP mRNA **(a)**. Detailed information on the primers sequences is provided in Supplementary Table S6. Histograms represent the average ± standard deviation of expression levels normalized to RPLP0 of two technical replicates performed for each biological replicate of si-RNA treated cells. Western blotting analysis of RBP protein level in whole-cell lysates from d3-diff C25Cl48 cells transfected with control negative siRNA (si-ctrl) or RBP siRNA. Anti-ß-Tubulin was used as a loading control **(b)**.

Supplementary Fig. S2

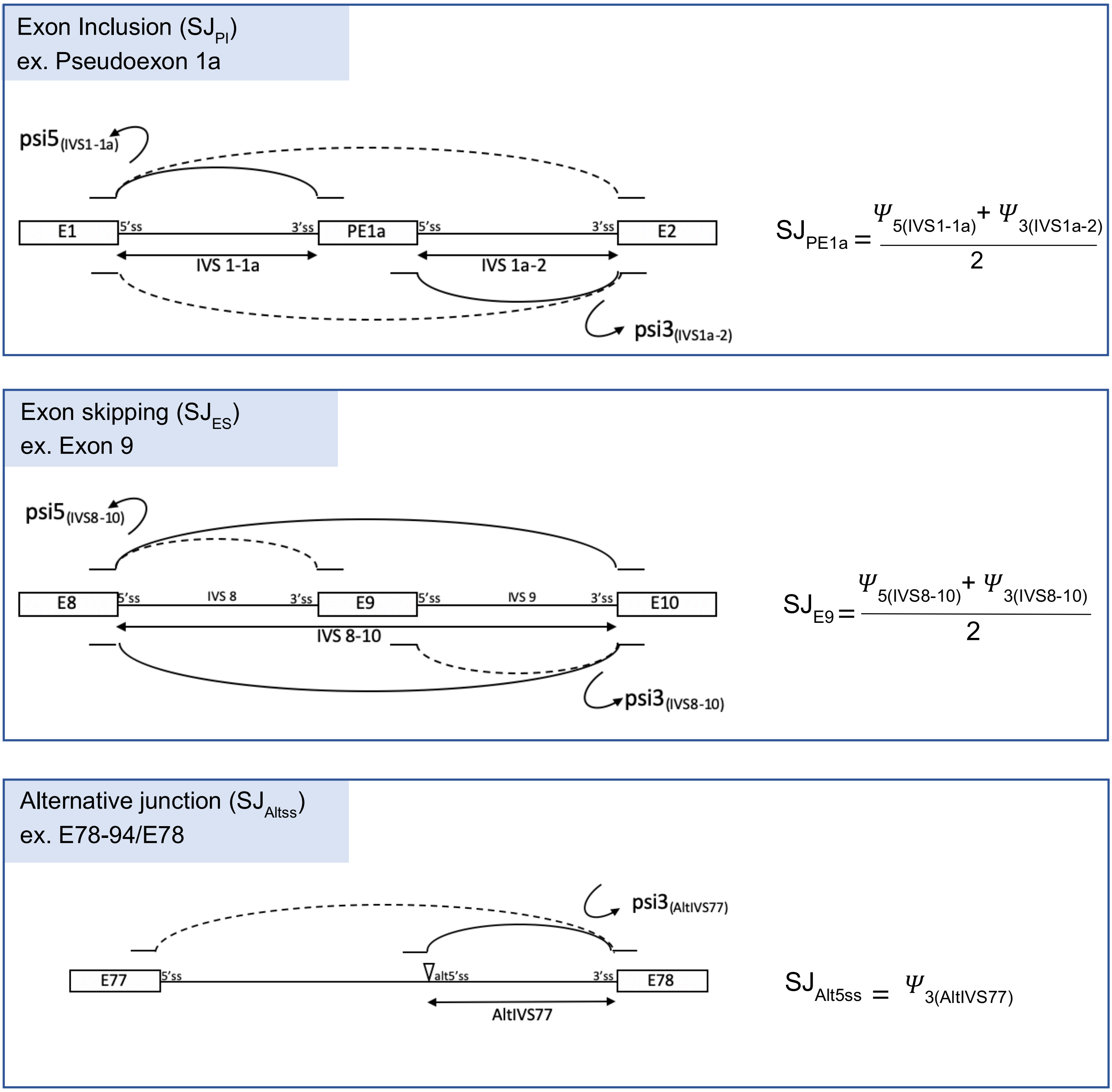

Supplementary Fig. S2 Calculation of novel Splice Junction (SJ) usage resulting from exon skipping (SJ_{ES}), exon inclusion (SJ_{PI}) or activation of alternative splice site (SJ_{Altss}) events according to the intron-centric metrics method (Pervouchine et al. 2013). Considering a specific intron, the psi5 means how often is the donor site (5' splice site, 5'ss) used with the acceptor site (3' splice site, 3'ss), compared to all other acceptors. As the same, psi3 means how often is the acceptor site used with the donor site, compared to all other donors.
