# Supplementary material for: First Identification of RNA-Binding Proteins That Regulate Alternative Exons in the Dystrophin Gene
Source: Int J Mol Sci. 2020 Oct 21;21(20):7803. doi: 10.3390/ijms21207803 (PMC7589424; doi:10.3390/ijms21207803)
Supplement: Supplementary file 1 [file ijms-21-07803-s001.zip › supplemental data/ijms-958132-supplementary-eng edited.docx]

**Legends to Supplementary Tables**

**Table 1.** Lists and sequences of siRNAs and primers used in this study.

**Table 2.** Alternative Splicing Events (ASEs) detected by *DMD*-targeted RNA-Seq in C25Cl48

cells differentiated for 3 days. List of ASEs in descending order of the percentage detected by

DMD-targeted RNA-seq in C25Cl48 cells at day 3 of differentiation (d3-diff) and their

respective values in skeletal muscle as reported in (**a**) (Bougé et al. 2017). In these experiments

(four biological replicates each), only new junctions covered by a minimum of 5 reads in at

least 2 out of the 4 replicates were considered. ND denotes “not determined” when the

minimum filtering criteria (new junction with ≥ 5 reads in at least 2/4 of the replicates) were

not reached but the number of reads is different from zero. The values of Alternative Splicing

Events (ASEs) are derived from the Splice Junction (SJ) usage provided by the SJPIPE

pipeline for pseudoexon (PE) inclusion (ASE (%) = SJPI × 100), for exon skipping events (del)

(ASE (%) = SJES × 100) and for use of alternative 3’ splice sites (3’ss) (in brackets, number of

exonic nucleotides deleted when the alternative 3’ss is used) (see Supplementary Fig.1 and

Materials and Methods for more details of the calculation).

**Table 3.** Gene expression microarray data in d3-diff C25Cl48 cells and in skeletal muscle.

For microarray analysis, total RNA samples from three human skeletal muscles (Myobank

20316, Clontech ref#636534, lots #1404229A and #1406360A) and three biological replicates

of C25Cl48 cells differentiated at day 3 were processed, hybridized on a GeneChip® Human

Gene 2.0 ST Array, scanned, and quantified at the Affymetrix Service Provider and Core

Facility, ‘‘KFB – Center of Excellence for Fluorescent Bioanalytics’’ (University of

Regensburg, Regensburg, Germany; www.kfb-regensburg.de). Briefly, RMA normalization

and Student’s *t*-test were used for statistical analysis. Benjamini–Hochberg correction for

multiple testing was applied in all tests and corrected *P*-values (FDR) < 0.05 were considered

significant.

GO 0008380 sheet: gene expression microarray data for the 305 genes analysed related to RNA

splicing (GO:0008380). The 18 genes with a up-(log2 fold-change of expression level (FC > 2))

or down-(FC < − 2) regulation in d3-diff C25Cl48 cells *versus* skeletal muscle tissue (False

Discovery Rate (FDR) < 0.05, two-Sample Student's *t*-tests) are highlighted in a different color.

FDR < 0.05 and FC < −2 or > 2 sheet: list of the genes that display statistically significant

regulation in d3-diff C25Cl48 cells *versus* skeletal muscle tissue (FC > 2 or FC < −2, FDR < 0.05,

two-Sample Student's *t*-tests). Over the 31,650 coding transcripts that were explored, a total of

2,605 (8.2 %) corresponding to 2,515 genes showed differential regulation, of which, 1,557

were up-(FC > 2) and 958 were down-(FC < −2) regulated.

**Table 4.** Quality assessment of targeted RNA-seq data.

RNA-seq read mapping sheet: the total number of reads mapping to the X-chromosome is

provided for each sample as well as uniquely mapped, multi-mapped and unmapped reads.

RNA-seq read counts sheet: the average read depth calculated from the total number of uniquely

mapped reads and the average mapped length obtained for each sample is given.

**Table 5.** DMD-targeted RNA-seq data: differential usage of splice junctions |ΔSJ| ≥ 0.05.

|ΔSJ|≥ 0.05 sheet: data from STAR mapping (reads) and Integrative Pipeline for Splicing

Analyses (IPSA) package (psi5, psi3) are given for each Splice Junction (SJ) found

differentially used at more than 5 % (|ΔSJ| ≥ 0.05) between the si-RBP and si-control

conditions. Data are given for each replicate of the si-ctrl (#1 to #4, line highlighted in grey) or

the si-RBP (#1 and #2) treated cells. The usage of the SJ is determined by the mean of psi5 and

psi3 values (see Supplementary Figure 1 for the calculation details depending on the type of

splicing events). Change in SJ usage is calculated as follows: △SJ = SJ_(si-RBP)_ − SJ_(si-ctrl)_.

PSI exon sheet: Percent spliced in index (PSI) of RBP-responsive exons. The PSI values

provided by the IPSA pipeline indicates the efficiency of splicing a specific exon into the

transcript population of a gene (exon relative usage). Change in exon usage between conditions

are calculated as follows: △PSI = PSI_(si-RBP)_ − PSI_(si-ctrl)_.

**Table 6.** DMD-targeted RNA-seq data: differential usage of splice junctions 0.05 ≥|ΔSJ| ≥ 0.01.

Data from STAR mapping (reads) and Integrative Pipeline for Splicing Analyses (IPSA)

package (psi5, psi3) are given for rare splicing events corresponding to differentially used

Splice Junction (SJ) in the interval of 0.05 ≥|ΔSJ|≥ 0.01 between the si-RBP and si-control

conditions. Data are given for each replicate of the si-ctrl (#1 to #4, line highlighted in grey) or

the si-RBP (#1 and #2) treated cells. The usage of the SJ is determined by the mean of psi5 and

psi3 values (see Supplementary Figure 1 for the calculation details depending on the type of

splicing events). Change in SJ usage is calculated as follows: △SJ = SJ_(si-RBP)_ − SJ_(si-ctrl)_.


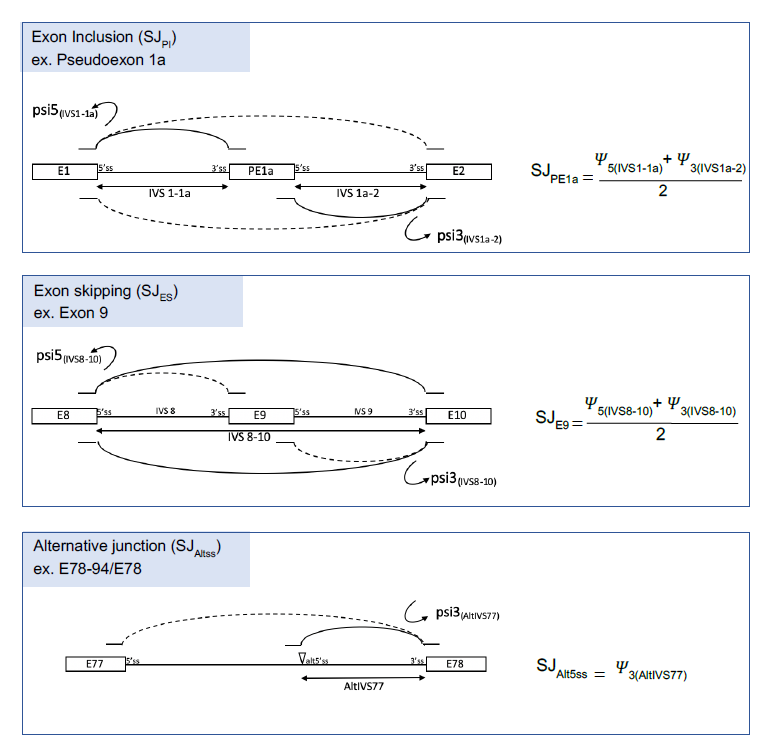


**Supplementary figure 1.** Calculation of novel Splice Junction (SJ) usage resulting from exon skipping SJES), exon inclusion (SJPI) or activation of alternative splice site (SJAltss) events according to the introncentric metrics method (Pervouchine et al. 2013). Considering a specific intron, the psi5 means how often is the donor site (5’ splice site, 5’ss) used with the acceptor site (3’ splice site, 3’ss), compared to all other acceptors. As the same, psi3 means how often is the acceptor site used with the donor site, compared to all other donors.

**Supplementary figure 2.** Assessment of siRNA-induced silencing of gene expression of the.

selected RNA-Binding Proteins (RBPs). RT–qPCR analysis of the relative mRNA level of

RBPs in d3-diff C25Cl48 cells transfected with either the control negative siRNA (si-Ctrl) or

the specific siRNA showing a generally marked decrease of the target RBP mRNA **(a)**. Detailed

information on the primers sequences is provided in Supplementary Table 1. Histograms

represent the average ± standard deviation of expression levels normalized to RPLP0 of two

technical replicates performed for each biological replicate of si-RNA treated cells. Western

blotting analysis of RBP protein level in whole-cell lysates from d3-diff C25Cl48 cells

transfected with control negative siRNA (si-ctrl) or RBP siRNA. Anti-ß-Tubulin was used as a

loading control **(b)**.
